# Novel Strategy of Curettage and Adjuvant Microwave Therapy for the Treatment of Giant Cell Tumor of Bone in Extremities: A Preliminary Study

**DOI:** 10.1111/os.12865

**Published:** 2021-01-13

**Authors:** Jin Ke, Shi Cheng, Meng‐yu Yao, Xiao Chu, Ming Wang, Xiao‐long Zeng, Tao Yang, Chi Zhang, Hua Zhong, Yu Zhang

**Affiliations:** ^1^ Department of Orthopaedics Guangdong Provincial People's Hospital, Guangdong Academy of Medical Sciences Guangzhou Guangdong China; ^2^ Department of Orthopaedics, Guangdong Key Laboratory of Orthopaedic Technology and Implant Materials General Hospital of Southern Theater Command Guangzhou Guangdong China; ^3^ Department of Orthopaedics The Fifth Affiliated Hospital, Southern Medical University Guangzhou Guangdong China

**Keywords:** Giant cell tumor, Microwave ablation, Distal radius, Pathological fracture

## Abstract

**Objectives:**

To evaluate whether curettage with adjuvant microwave therapy was successful in the treatment of giant cell tumor of the bone (GCTB) in extremities, especially for GCTB with pathological fractures and GCTB of the distal radius.

**Methods:**

This was a retrospective study of 54 cases of GCTB of the extremities treated by curettage with adjuvant microwave therapy between 2007 and 2019. Five patients were lost to follow up and excluded from the study. A total of 33 male and 21 female patients were included in this study. Patients were aged 15–57 years (mean 29.72 ± 10.48 years). Among these patients, there were 10 cases of GCTB with pathological fractures and eight cases of GCTB of the distal radius; one of these cases was combined with a pathological fracture. Comprehensive imaging examinations (X‐rays [including lesion site and chest], CT, MRI, emission computed tomography, and pathology examination) of all patients were reviewed. The clinical staging of these patients were evaluated radiologically using the Campanacci classification system based on the extent of spread of the tumor. All patients underwent curettage with adjuvant microwave therapy. Clinical and imaging evaluations were performed in all cases to check for recurrence or metastasis. Lower limb and upper limber function were assessed using the Musculoskeletal Tumor Society score (MSTS), and wrist function was assessed according to the disabilities of the arm, shoulder and hand (DASH) score. Data on surgical‐related complications were recorded.

**Results:**

All cases were followed up for 24–126 months (mean 60.69 ± 29.61 months). There were 24 patients with a Campanacci grade of 3 and 30 with a Campanacci grade of 2. The 52 patients were continuously disease‐free. The local recurrence rate was 3.70% (2 patients). One patient had recurrence in the proximal femur, and the other developed in soft tissue of the calf muscle. No recurrence occurred for GCTB of the distal radius. One recurrence occurred in a GCTB with pathological fractures. The intervals were 9 and 28 months, respectively. The cases of recurrence all had a Campanacci grade of 3 (8.33%). The median MSTS among the 54 patients was 27.67 ± 3.81. The mean wrist function DASH score was 8.30 ± 2.53. The mean MSTS was 28.67 ± 1.63 and 26.71 ± 5.49 for patients with GCTB of the distal radius and for those with pathological fractures, respectively. In comparing patients with and without pathological fractures, there was no significant difference in the MSTS functional score. Five patients had complications after the surgery.

**Conclusion:**

Curettage with adjuvant microwave ablation therapy provided favorable local control and satisfactory functional outcomes in the treatment of GCTB, especially for cases with pathological fractures and those with GCTB of the distal radius.

## Graphical abstract

Schematic diagrams of giant cell tumor in proximal tibia are shown to illustrate the heating and monitoring. (A) Microwave ablation probe. (B) Temperature probe in knee joint, adjacent to the lesion. (C) Flow of cryogenic saline cools the articular cavity to protect the normal structure, including cartilage, meniscus, and cruciate ligament. (D) Gauze soaked with ice brine isolates the vascular and nerve bundle from the tumor lesion. Sometimes we placed our hand between the tumor lesion and the important vascular nerve to prevent damage to the vascular nerve. The orange region represents the heating range of the microwave.

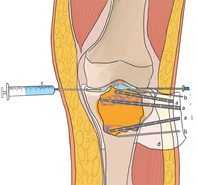

High fever may cause damage to normal surrounding tissue. a series of protective measures were taken during the operation. the gauze soaked with ice brine isolates the posterior vascular and nerve bundle, the cooled sterilized water was injected into the knee joint to protect the the structure in the knee joint. In the meantime, continuous multi‐point temperature measurement can play a important role in monitoring.

## Introduction

Giant cell tumors of the bone (GCTB) are primary intramedullary bone tumors composed of mononuclear and giant mononuclear cells similar to osteoclasts, which present as locally aggressive lesions with unpredictable behavior^1^. GCTB account for approximately 5% of primary bone tumors and 20% of all benign tumors^2^. The occurrence of GCTB is primarily observed in individuals between 20 and 40 years of age. The site of predilection for GCTB is the extremities of long bone in skeletons of mature adults. Due to its unpredictable tendency to recur locally and to potentially metastasize, GCTB is considered to have a low malignant potential^3^. GCTB patients can survive for a long time after appropriate treatment; therefore, the impact of surgical treatment on limb function should be fully considered. The choice of surgical method depends on many factors, such as tumor size and the existence of pathological fractures, bone cortex damage, soft tissue involvement, or articular cartilage damage^4^, ^5^. Intralesional excision with curettage is the standard method of treatment, but this has been associated with local recurrence rates ranging from 10% to 40%^2^, ^6^. Wide resection may increase the recurrence‐free survival rate to 84% to 100%^7^, ^8^, ^9^. However, the wide resection is associated with higher rates of surgical complications and is accompanied by considerable functional impairment^10^. Determing the ideal clinical treatment for GCTB is still a challenge to the orthopaedic oncologist, especially for some types such as GCTB of the distal radius and GCTB with pathological fractures.

The GCTB of the distal radius account for approximately 10% of the total types of GCTB^11^. Treatment options have included intralesional excision (curettage) with or without adjunctive modalities (e.g. high‐speed burring, cryotherapy, phenol, and hydrogen peroxide). Cryosurgery adjunct to curettage can preserve the distal radius and its normal articulations^12^. It allows eradication of residual tumor cells from under the subchondral bone and subperiosteal covering, where curettage obviously cannot be completely effective. However, cryosurgery freezes and damages surrounding normal tissue as it kills the residual tumor cells. This can translate into higher complications rates, which can negate the benefit of improved tumor control^11^. Another treatment option is en bloc resection followed by reconstruction, or arthrodesis^13^. Because of the more aggressive behavior and limited surrounding soft tissue of GCTB of the distal radius compared with GCTB in other parts, the clinical recurrence rate is higher than for other types of GCTB after initial simple intralesional curettage treatment^14^. Recurrence rates have ranged from 25% to 89%^15^, ^16^. In addition, en bloc resection usually requires sacrifice of the articular surface, and secondary arthritis has occurred in 13% to 50% of patients^17^.

The incidence of GCTB with pathological fractures is approximately 9% to 30%^2^. Because the local soft tissue may have been contaminated by tumor cells, there is still a high recurrence rate even after enlarged intracapsular curettage, and most local recurrences occur within the first 2 years postoperatively^18^. The use of curettage with adjuvants is associated with relatively high local recurrence rates (12%–34%)^7^, ^19^. Some authors consider resection and reconstruction the preferred treatment in patients with severe joint destruction or intraarticular fractures^20^. Although the risk of local recurrence is generally low after en bloc resection (0%–12%)^8^, ^21^, ^22^, it is not necessarily the most favorable primary treatment.

For the two types of GCTB mentioned above, surgeons are more inclined to select en bloc resection. However, in using this method, the problem of postoperative functional deficit needs to be considered when the sacrifice of periarticular structures is required for the resection and reconstruction, especially for young adults aged 20 to 40 who account for most GCTB patients^17^, ^23^, ^24^. Therefore, there is an urgent need to investigate effective treatments that could both reduce the recurrence rate and maintain the extremities’ function for these types of GCTB.

Adjuvant treatments such as high speed burring, cryotherapy, phenol, and hydrogen peroxide have been recommended for reducing recurrence rates of GCTB. These types of adjuvant therapy have been extensively used in clinic, and the reported recurrence rate ranges from 0% to 28%^8^, ^18^, ^25^. Microwave ablation, which could rapidly generate high temperatures to destroy tumor tissues, has been widely adopted in the treatment of bone tumors, such as osteosarcoma, osteoid osteoma, and metastatic tumors of the bone^26^, ^27^, ^28^. This approach makes it possible to preserve more native joints when performing limb salvage surgery for bone tumors. Although microwave adjuvant therapy has been proven to be a safe and effective method to treat many kinds of bone tumors^29^, ^30^, ^31^, there are no studies focusing on the application of this method in the treatment of GCTB in extremities, especially for the two types mentioned above.

The aims of this study were to investigate: (i) whether curettage with adjuvant microwave ablation therapy is safe for the treatment of GCTB in the extremities; (ii) whether curettage with adjuvant microwave ablation therapy can reduce the recurrence rates of GCTB with pathological fractures and GCTB of the distal radius; and (iii) whether patients who undergo curettage with adjuvant microwave ablation therapy have satisfactory limb function, especially for GCTB with pathological fractures and of the distal radius.

## Patients and Methods

### *Patients Selection*

The inclusion criteria for the present study were: (i) patients with GCTB in the extremities and follow‐up time of more than 2 years; (ii) patients who had received curettage with adjuvant microwave therapy between 2007 and 2019; (iii) the main evaluation indicators included oncological outcome and the function outcome of extremities; and (iv) a retrospective study. The exclusion criteria were as follows: (i) patients with GCTB in the spine and follow‐up time of less than 2 years; and (ii) patients do not received microwave ablation adjuvant curettage therapy.

A total of 59 GCTB of the extremity patients underwent curettage with adjuvant microwave therapy; 5 patients were lost to follow‐up. There were 18 (33.33%) patients with either of the two types considered in this study. Some recieved primary treatment at our hospital and others were referred to our hospital with local recurrence after treatment elsewhere; the clinic data of these patients were retrospectively collected. Among the 18 patients, there were 10 with pathological fractures. Of the 8 patients with GCTB of the distal radius, 1 case was combined with a pathological fracture, and one was a recurrent GCTB. This study was approved by our institutional review board. All procedures performed in this study were in accordance with the ethical standards of the institutional and/or national research committee and with the 1964 Declaration of Helsinki and its later amendments or comparable ethical standards.

### *Data Collection*

Comprehensive imaging examinations (X‐ray [including lesion site and chest], CT, MRI, emission computed tomography, and pathology examination) of all patients were reviewed. The clinical staging of these patients were evaluated radiologically by two experienced radiologist and two oncologists using the Campanacci classification system based on the extent of spread of the tumor. The percentage of bone occupied by the tumor was calculated as the proportion of the cross‐sectional area of the bone at the widest dimension of the tumor, as in Prosser *et al*. (2005)^32^. All patients provided written informed consent for their data to be included in this study.

### *Surgical Procedures*

Surgical procedures were the same as for conventional extensive curettage procedures except that before the ablation the tumor bone and invaded soft tissue were separated from the surrounding normal tissue by gauzes, which was especially important for cases with pathological fractures. Then the microwave antenna array was inserted into the tumor segment evenly for ablation. Microwave ablation was performed with an MWA system (2450 MHz, MTI‐5A, Great Wall, Nanjing, China).

#### *Microwave Ablation Technique*

**Fig 1:**
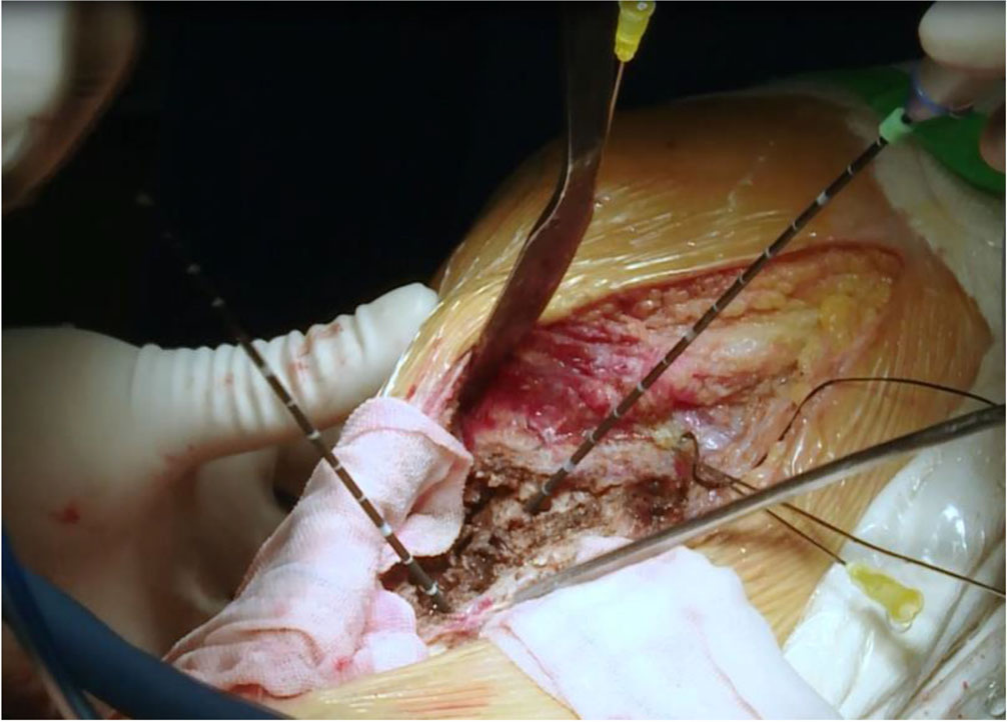
An intraoperative photo of a giant cell tumor of the bone (GCTB) in the proximal tibia. The microwave antennas’ shaft positions were repositioned between ablation cycles. The temperature of the articular cartilage and ligaments was continuously monitored by thermometry needles, and cooled sterilized water was injected into the knee joint to ensure that the temperature was below 43°C. The gauze soaked with ice brine isolates the vascular and nerve bundle from the tumor lesion.

The microwave antenna shaft position was repositioned between ablation cycles to obtain a larger thermo ablation zone and positioned at the edge of the lesion to achieve a larger ablation margin by controlling the power (temperature measurement confirmed), which is shown in schematic diagrams. An illustrative case of the intraoperative procedure is shown in Fig. 1. The ablation range was more than 2 cm beyond the boundary of the tumor tissue, in accordance with to Li *et al*. (2015)^29^. On the articular side, if the subchondral bone was less than 1 cm, there was no need to meet the above requirements. Two syringes were inserted simultaneously into the joint cavity and continuously monitored by thermometry needles. A flow of cryogenic saline cooled the articular cavity to protect the normal structure (e.g. cartilage, meniscus, and cruciate ligament). The cooled sterilized water was applied to ensure that the temperature of adjacent normal tissue was below 43 °C. Occassionally, we placed our hand between the tumor lesion and the important vascular nerve to prevent damage to the vascular nerve.

#### *Curettage after Ablation*

**Fig 2:**
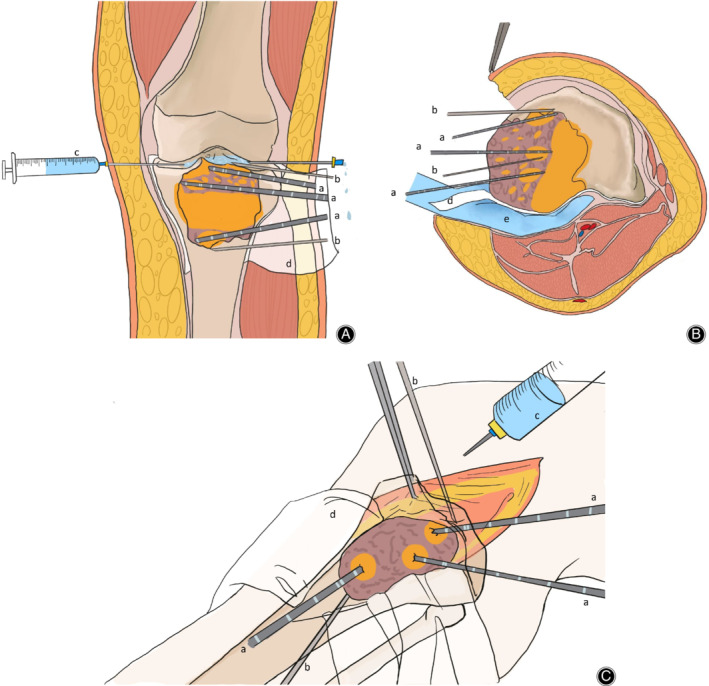
Schematic diagrams (A‐C) of a giant cell tumor in the proximal tibia illustrate the heating and monitoring from three different perspectives. When we ablate the lesion adjacent to the articular surface, we need to pay attention to protect the structure in the articular cavity, and inject ice brine into the articular cavity.Meanwhile, Attention should be paid to the protection of the posterior vascular nerves when we ablate the lesions of adjacent vessels. such as Gauze soaked with ice brine or our hand.(a) Microwave ablation probe. (b) Thermometry needle in the knee joint and adjacent to the lesion. (c) Flow of cryogenic saline cools the articular cavity to protect the normal structure, such as cartilage, meniscus, and cruciate ligament. (d) Gauze soaked with ice brine isolates the vascular and nerve bundle from the tumor lesion. (e) Occassionally, we placed our hand between the tumor lesion and the important vascular nerve to prevent vascular nerve damage. The orange region represents the heating range of the microwave.

After the ablation process is complete, the length of the window should be 10 mm larger than the tumor in the longitudinal direction and the width should be at least one‐third larger than the perimeter in cross‐section. The intraoperative ablation and monitoring are illustrated in Fig. 2. The necrotic tumor tissues were completely scraped off. The residual debris was completely washed away by sterilized water. The defected part of the tumor cavity was filled with bone cement or allogeneic bone.

### *Postoperative Follow Up*

Routine follow‐up examinations were conducted every 4 months for the first 2 years, every 6 months for the next 3 years, and then annually. All cases were followed up regularly to observe any local recurrence, malignant transformation, or distant metastasis. Both radiological images and medical records were collected at each follow‐up. The function outcomes of upper and lower limbs were evaluated according to the Musculoskeletal Tumor Society score. All the data collection was conducted by a group of independent doctors. The treating surgeons were blind to this data.

### *Evaluation Index*

#### *Musculoskeletal Tumor Society*

The Musculoskeletal Tumor Society score (MSTS) developed by Enneking was used to assess the functional results^33^. The MSTS mainly includes six aspects: pain, function, emotional acceptance, supports, walking, and gait. The score standard had a maximum of 30 points (best possible outcome).

#### *Disability of Arm, Shoulder and Hand Instruction Score*

The Disability of Arm Shoulder and Hand Instruction (DASH) score was used to assess the functional results of the wrist after the surgery. The DASH score mainly includes 23 aspects. The score standard had a minimum of 23 points (best possible outcome) and a maximum of 115 (worst possible outcome).

#### *Recurrence‐Free Survival*

The recurrence‐free survival was defined as the interval between the first surgery and signs of recurrence by imaging during follow‐up.

### *Statistical Analysis*

SPSS24.0 (SPSS, Chicago, IL, USA) was used to analyze the data. Recurrence‐free survival was evaluated by the Kaplan–Meier method. χ^2^ analysis was performed to estimate the difference in various percentages. A *P*‐value <0.05 was considered statistically significant.

## Results

### *Patient Demographics and Surgical Data*

A total of 54 patients were included in this research. The mean age of patients was 29.72 ± 10.48 years (15–57 years). The median follow up was 60.69 ± 29.61 months (24–126 months). The characteristics of patients are summarized in Table 1.

**TABLE 1:** General information of patients [cases (%)]

Variables	Number of patients (*n* = 54)	Without local recurrence (*n* = 52)	With local recurrence (*n* = 2)	Without complication (*n* = 49)	With complication (*n* = 5)
Gender					
Male	33 (61.11)	33 (61.11)	1 (1.85)	30 (55.56)	4 (7.27)
Female	21 (38.89)	20 (37.04)	1 (1.85)	20 (37.04)	1 (1.82)
Site					
Distal femur	9 (16.67)	9 (16.67)	0 (0)	9 (16.67)	0 (0)
Proximal tibia	14 (25.93)	14 (25.93)	0 (0)	13 (24.07)	1 (1.85)
Distal radius	8 (14.81)	8 (14.81)	0 (0)	7 (12.96)	1 (1.85)
Proximal femur	8 (14.81)	7 (12.96)	1 (1.85)	6 (11.11)	2 (3.70)
Distal tibia	2 (3.70)	1 (1.85)	1 (1.85)	1 (1.85)	1 (1.85)
Huckle‐bone	3 (5.56)	3 (5.56)	0 (0)	3 (5.56)	0 (0)
Calcaneus	2 (3.70)	2 (3.70)	0 (0)	2 (3.70)	0 (0)
Patella	3 (5.56)	3 (5.56)	0 (0)	3 (5.56)	0 (0)
Proximal humerus	5 (9.26)	5 (9.26)	0 (0)	5 (9.26)	0 (0)
Combined pathological fractures	8 (14.81)	8 (14.81)	1 (1.85)	7 (12.96)	1 (1.85)
Campanacci classification					
Stage 1	0 (0)	0 (0)	0 (0)	0 (0)	0 (0)
Stage 2	30 (55.56)	30 (55.56)	0 (0)	29 (53.70)	1 (1.85)
Stage 3	24 (44.44)	23 (42.59)	2 (3.70)	20 (37.04)	4 (7.41)
Previous surgery					
None	49 (90.74)	49 (90.74)	1 (1.85)	45 (83.33)	4 (7.41)
One	5 (9.26)	4 (7.41)	1 (1.85)	4 (7.41)	1 (1.85)

### *Postoperative Evaluation*

Based on the results obtained, the three questions posed by this study are answered in what follows.

#### *Recurrence‐Free Survival*

**Fig 3:**
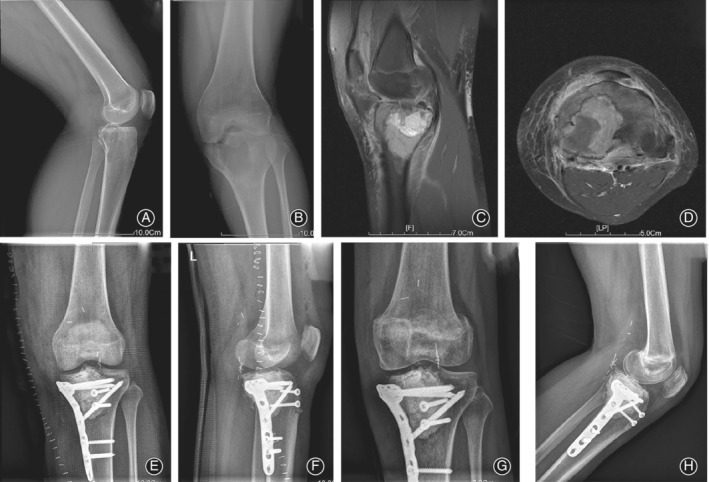
An illustrative case of giant cell tumor of the bone (GCTB) with pathological bone fracture. (A, B) Preoperative frontal and lateral X‐rays of the knee, diagnosed with proximal tibial GCTB with pathological fracture. (C, D) Preoperative MRI images of the knee, which indicated the extent of the tumor, Campanacci grade III. (E, F) Postoperative frontal and lateral X‐rays of the knee. During the operation, the ligamentum patellae was cut off and protected and was reconstructed *in situ* after microwave ablation. (G, H) Postoperative frontal and lateral X‐rays after 2 years; no local recurrence was observed.

**Fig 4:**
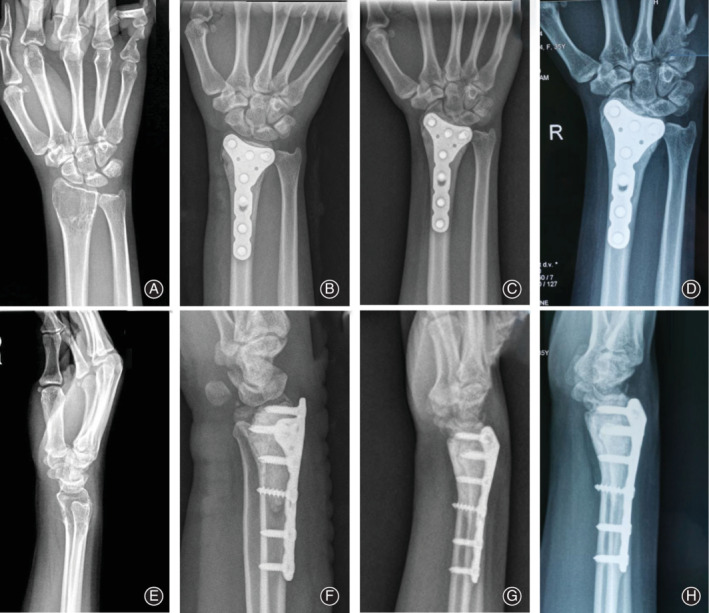
An illustrative case of giant cell tumor of the bone (GCTB) of the distal radius. (A, E) This radiograph shows a GCTB of the distal radius, Campanacci II. (B, F) Postoperative frontal and lateral X‐rays; bone cement filled the cavity after curettage. (C, G) 4 years after the operation, no local recurrence was observed. (D, H) 5 years after the operation, there was no local recurrence.

Oncological outcomes showed that 52 patients were continuously disease‐free. Two (3.70%) of the 54 patients developed a local recurrence after a median of 18.5 months (9 and 28 months respectively). One had recurrence in bone (the primary site is the proximal femur); the interval between the first surgical treatment and local recurrence was 9 months. Treatment of the local recurrence was also curettage combined with microwave ablation; there was no recurrence at the 4‐year follow up. There was 1 case of a distal tibial GCTB with a pathologic fracture. Recurrence occurred in soft tissue of the calf muscle (the primary site was the distal tibia). The interval was 28 months, and the patient received lesionectomy. No patient with distal radius GCTB had local recurrence after surgery at the last follow up (0%). Local recurrence occurred in 2 patients with a Campanacci grade of 3 (8.33%). No patient had lung metastasis. Illustrative cases of each type of GCTB are shown in Figs. 3 and 4.

**Fig 5:**
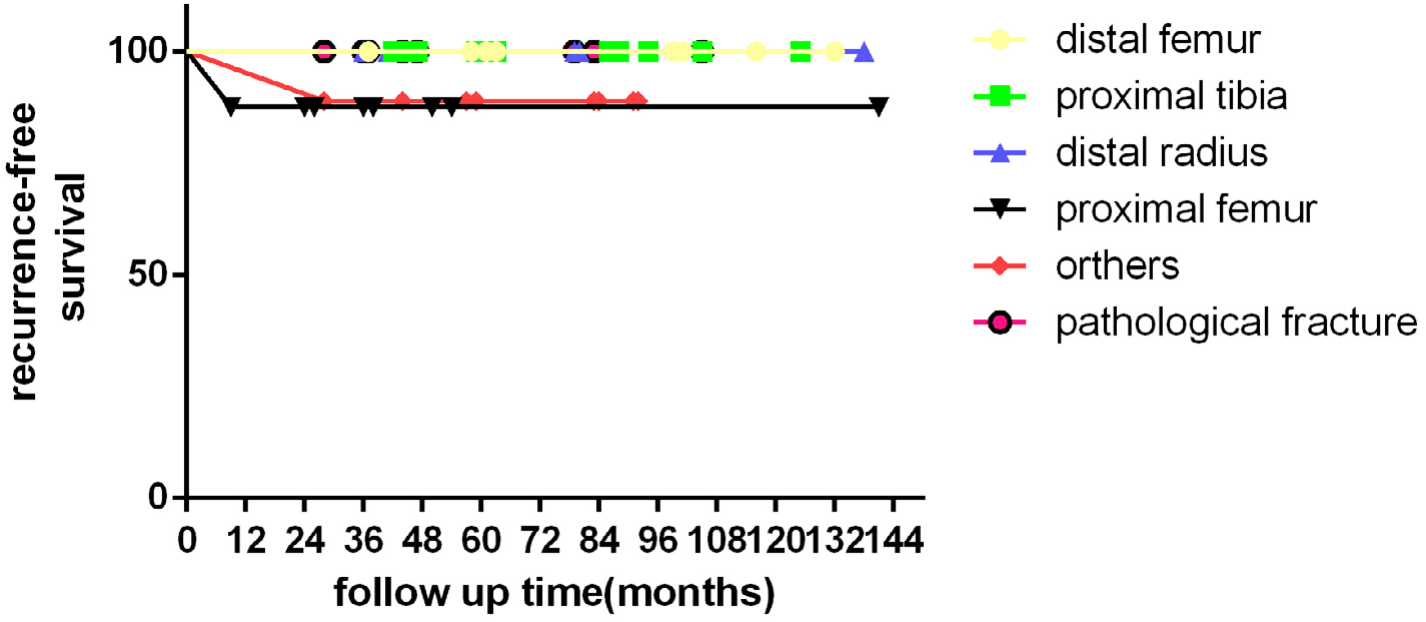
Recurrence‐free survival by site in 54 giant cell tumors of the bone of the long bones (distal radius, distal femur, proximal tibia, proximal femur, other sites, and with pathological fracture).

Univariate analysis revealed that no significant statistical effect on the local recurrence rate could be identified for gender, Campanacci stage, lesion site, and presence or absence of pathological fracture (Table 2). The Kaplan–Meier curve of the cumulative survival is shown in Fig. 5, with local recurrence as the end point, according to the different sites and two types of GTCB based on 54 cases. The differences among the sites and the two types were not significant (Fig. 5). Although a limited number of patients were enrolled, we believe that microwave adjuvant therapy could play a role in reducing the recurrence rate of GCTB, especially for GCTB of the distal radius and those with pathological fractures. The corresponding MSTS and DASH scores are shown in Table 3.

**TABLE 2:** Univariate analysis for recurrence‐free survival

Variables	Recurrence‐free (*n* = 52)	Recurrence (*n* = 2)	Total (*n* = 54)	*P*‐value
Recurrence preoperation				0.649
No	47 (87.0%)	2 (100.0%)	49 (90.7%)	
Yes	5 (9.2%)	0 (0.0%)	5 (9.3%)	
Pathological fracture				0.235
No	43 (79.6%)	1 (50.0%)	45 (83.3%)	
Yes	9 (16.7%)	1 (50.0%)	10 (18.5%)	
Age				0.567
Mean (standard deviation)	30.755 (10.336)	26.500 (3.536)	30.600 (10.186)	
Range	13.000–57.000	24.000–29.000	13.000–57.000	
Gender				0.726
Male	32 (59.3%)	1 (50.0%)	33 (61.1%)	
Female	20 (37.0%)	1 (50.0%)	21 (38.9%)	
Site				0.494
Proximal femur	7 (13.2%)	1 (50.0%)	8 (14.5%)	
Distal femur	9 (17.0%)	0 (0.0%)	9 (16.4%)	
Proximal tibia	14 (28.3%)	0 (0.0%)	14 (27.3%)	
Others	14 (26.4%)	1 (50.0%)	15 (27.3%)	
Distal radius	8 (15.1%)	0 (0.0%)	8 (14.5%)	
Campanacci classification				0.115
2	30 (56.6%)	0 (0.0%)	30 (54.5%)	
3	22 (43.4%)	2 (100.0%)	24(45.5%)	

**TABLE 3:** Functional evaluation of GCTB of the distal radius and GCTB with pathological fractures

Functional evaluation	Distal radius	Pathologic fracture
Mean MSTS (last follow up)	28.67 ± 1.63	28.40 ± 2.19
Mean DASH score (last follow up)	8.30 ± 2.53	**/**
Disease free survival	100%	90%
Local recurrence	None	1

DASH, disabilities of the arm, shoulder and hand; GCTB, giant cell tumor of the bone; MSTS, Musculoskeletal Tumor Society score.

#### *Assessment of Limb Function*

**Fig 6:**
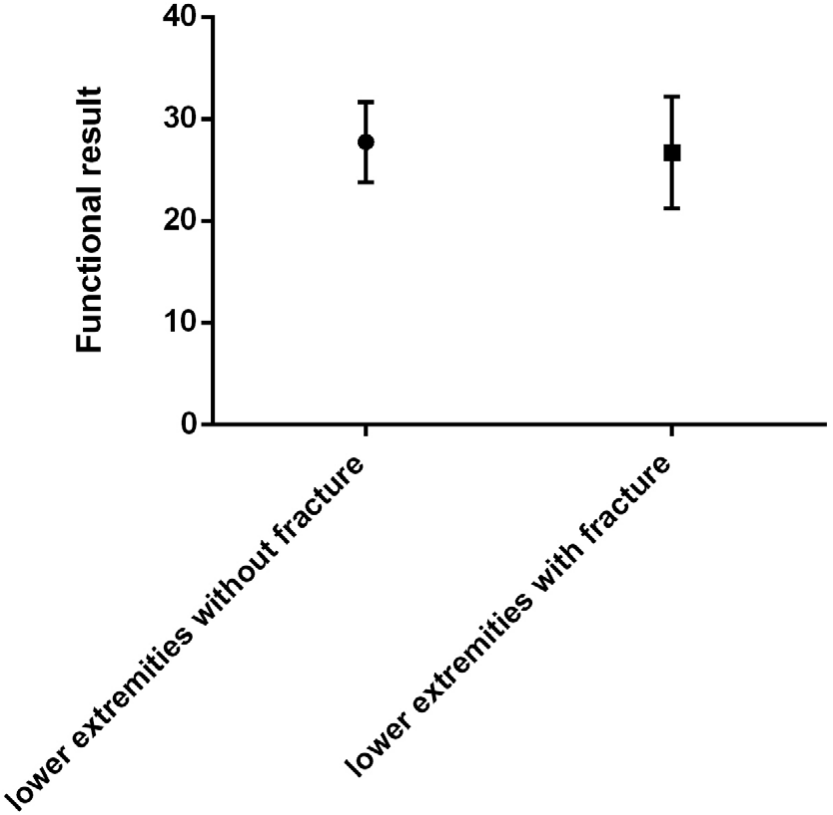
Mann–Whitney *U*‐test shows no significant difference in the Musculoskeletal Tumor Society score between lower extremities with and without pathological fractures.

The median MSTS of the 54 patients was 27.67 ± 3.81. The mean MSTS was 28.67 ± 1.63 and 26.71 ± 5.49 for patients with GCTB of the distal radius and GCTB with pathological fractures, respectively. The mean DASH score for GCTB of the distal radius was 8.30 ± 2.53. In comparing patients with and without pathological fractures, there was no significant difference in the MSTS functional score (Fig. 6).

#### *Surgical Safety Assessment*

**Fig 7:**
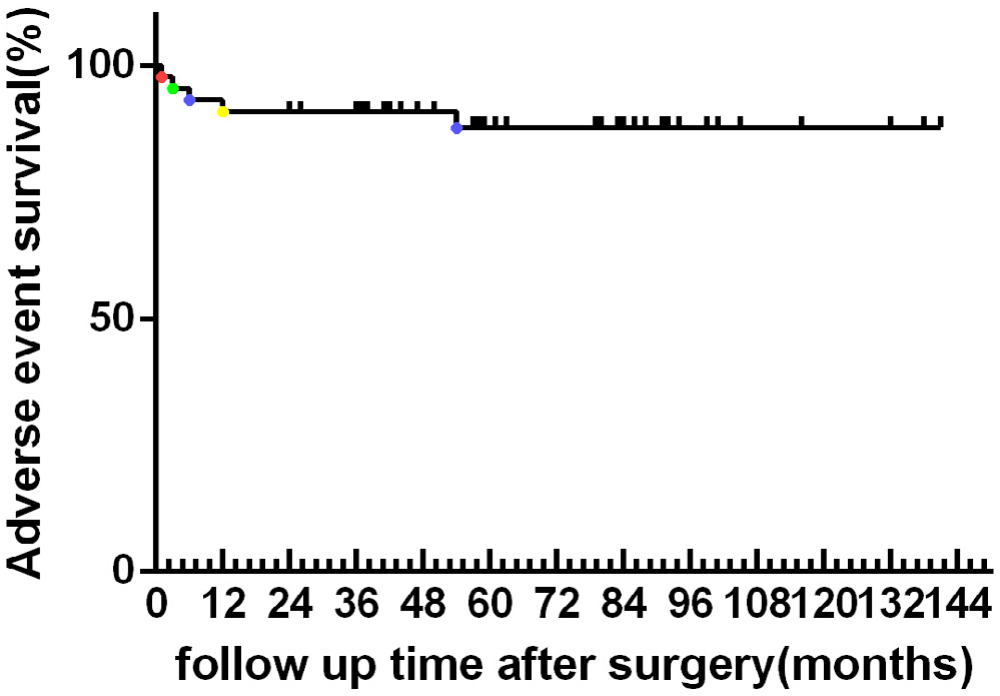
Adverse event survival by site in 54 giant cell tumor of the bone of the long bones. The dots in different colors represent the time of the adverse event. Red point: infection. Green point: subluxation. Blue point: femoral head necrosis. Yellow point: pathological fracture.

With regard to adverse events among the 54 patients, 5 patients (9.25%) had complication after the surgery. One patient experienced deep tissue infection 1 month after the operation (1.85%). The infection was uncontrollable after repeated debridement; therefore, we excised the infectious part of the bone and used the Masquelet technique to repair the bone defect. Two patients experienced osteonecrosis of the femoral head after the operation (3.70%): the interval between the first surgical treatment and femoral head necrosis was 6 months and 54 months, respectively. The first patient further showed subsiding of femoral head and hip joint subluxation 9 months after initial surgery. Another received resection with prosthesis. This patient was followed up without obvious symptoms. One patient had wrist joint subluxation after surgery (1.85%); the interval between the first surgical treatment and the complication of this patient was 3 months. At present, wrist flexion and prerotation activities of the patient are slightly limited. Fracture occurred in 1 patient 1 year of the surgery (1.85%); he then received resection with prosthesis. Special attention must be given to giant cell tumors of the proximal femur, where the treatment is associated with a relatively higher rate of osteonecrosis of the femoral head. Illustrative cases of adverse events are shown in Fig. 7.

## Discussion

Giant cell tumors of the bone have a strong tendency for local recurrence and the potential to metastasize to the lungs, especially GCTB with pathological fractures and of the distal radius^34^, ^35^. At present, the most commonly used method to treat GCTB is intralesional curettage and cement filling, with the reported local recurrence rates ranging from 8.6%–16%^21^, ^22^. However, this conventional approach may not be the optimal surgical treatment for these types of GCTB considering that these types are associated with a high rate of recurrence^21^, ^22^, ^36^. In view of the successful application of microwave ablation treatment in various bone tumors^26^, ^29^, curettage with adjuvant microwave ablation therapy was performed to treat GCTB in this study. The results of our study show satisfactory oncological and functional outcomes regardless of, for instance, the Campanacci stage and the tumor site.

### *Giant Cell Tumor of the Bone of the Distal Radius and Giant Cell Tumor of the Bone with Pathological Fractures*

At present, there is no consensus about the optimal surgical treatment for these two types of GCTB. For GCTB of the distal radius, previous studies reported that it is possible to treat these grade 3 lesions using a wide or marginal excision of the soft tissue mass, and then treat the residual intraosseous tumor, as a grade 1 or 2 lesion, by curettage and local adjuvant therapy to excise the contaminated tissue^37^. A custom unipolar wrist hemiarthroplasty has been suggested to treat GCTB of the distal radius; however, this method has several potential complications, such as progressive wrist joint degeneration^38^. Despite no difference in the grip strength and pinch strength, wrist fusion surgery was unacceptable for most patients due to loss of wrist motion^11^. For GCTB with pathological fractures, some scholars are concerned that surgical intervention will disseminate the tumor cells into the surrounding soft tissues and adjacent joints.^4^ Thus, Niu *et al*. suggested that GCTB with pathological fractures is one of the indications for en bloc resection^21^. Tsukamato *et al*. reported that the rate of local recurrence for these patients was 22.0% after curettage and 8.8% after resection, demonstrating the superior effects of en bloc resection in reducing the recurrence rate of GCTB^39^. Tumor prosthesis replacement is the common method for reconstruction after en bloc resection. Disappointingly, tumor prosthesis replacement after tumor resection has a high failure rate, of up to 58%^40^. However, some scholars have compared the postoperative recurrence rate of patients with GCTB with and without pathological fractures, and have found no significant difference between the two groups after intracapsular curettage^4^. In summary, although there is academic controversy about the optimal treatment for the two types of GCTB, we believe that the main reason for local recurrence is incomplete tumor resection. However, it is difficult for surgeons to determine whether or not tumor residual exists during surgery. Microwave ablation can generate high temperatures in a short time in the lesion. We believe this method could made tumor curettage more safe because the residual tumor tissue was devitalized after ablation. In this study, the results showed that there were no cases of recurrence or metastasis in GCTB of the distal radius and there was 1 case (1.85%) in GCTB with a pathological fracture. Although we only enrolled a small case series, the role of microwave ablation in preventing tumor recurrence was still impressive.

### *Advantage of Curettage with Adjuvant Microwave Ablation*

As a typical limb‐salvage and bone preservation technique, we believe that this method is more suitable for young patients because young patients have a long predicted survival time and desire maximal preservation of extremities for activity^26^. In our surgical procedures, the tumor was separated from the surrounding tissues. In the cases of pathological fractures, the tumor tissues were not incised, to prevent the tumor contaminating the surrounding tissues. To achieve a thorough ablation, the microwave needles were placed into the tumor bone for ablation according to the range of lesions measured by preoperative MRI. The interval of each needle was 1.5 cm, and the ablated range of the needle on the vertical axis was more than 2 cm beyond the normal boundary. We selected 2 cm as a safe distance because our previous study on animal specimens revealed that the ablation scope of bone specimens was larger than 3 cm, even at a minimal microwave power and time; the results are shown in the supplementary materials. Other studies have also used 2 cm as the ablation extent^26^, ^29^. In cases of lesions larger than 4 cm, the microwave antenna shaft position was repositioned between ablation cycles to obtain a larger thermo ablation zone^29^. At the same time, the thermometry needles were used to monitor the temperature of each position in the tumor in real time to ensure the center temperature of the tumor reached 100°C. The ablation of cortical bone and periosteum by microwave was more thorough, which can be used as a reason to explain the low recurrence rate of giant cell tumors in this site. In the only case with a pathological fracture that developed recurrence, it was speculated that the surrounding soft tissue was contaminated by the tumor. Recurrence in the 1 case of GCTB of the proximal femur was a result of insufficient ablation. In conclusion, we hold the view that satisfactory exposure and thorough ablation are critical to reduce the recurrence rate of these types of GCTB.

Another advantage of microwave ablation is the preservation of native joints, which show excellent function in the long term compared to prosthesis replacement. In Yang *et al*.^24^, in which knee joint tumor reconstruction patients were followed up for an average of 77 months, MSTS functional scores were greater than 66%, and the overall rating was excellent or good. According to Albergo *et al*., joint function is largely impaired after en bloc resection, and tumor prosthesis or allogeneic bone joint reconstruction cannot completely restore the original joint function of the patient^40^. Van der Heijden *et al*.^4^ treated 48 cases of GCTB with pathological fractures using intralesional curettage (23 cases) and en bloc resection (25 cases). The results showed that intralesional curettage provided better limb functional outcomes than tumor resection. Thus, the functional outcome was closely related to the integrity of joint preservation. Numerous scholars have recommended microwave ablation as treatment for bone tumors^26^, ^29^ because it can maintain the integrity of the joint and achieve biological repair of the bone defect lesion. Combined with an early and regular rehabilitation exercise program after surgery, patients would have an improved recovery after microwave ablation surgery^26^.

### *Complications*

A few complications were observed in this study, such as deep infection, ligament injury, femoral head necrosis, and fracture. One of the 54 cases followed up suffered a subluxation of the wrist joint 3 months after the operation due to sacrifice of periarticular structures of the wrist. One case of distal tibial was infected 1 month after surgery. The reason for this complication was considered to be less soft tissue and insufficient coverage around the lesion site. Meanwhile, the residual tumor necrosis and the possibility of intraoperative contamination could not be excluded. Two patients with GCTB of the proximal femur developed femoral head necrosis. Our suggested reasons for this complication are as follows. First, to achieve better exposure of the tumor lesion and to better protect the surrounding nerves and blood vessels from hyperthermia caused by microwave ablation, the joint capsule was cut open. Thus, the blood supply of the branches of the lateral femoral sac and the joint capsule was impacted, potentially further interfering with the blood supply of the femoral head. Second, during the microwave ablation process, the central temperature of the tumor was up to 100 °C^41^. The precise scope of microwave ablation was difficult to control. Furthermore, to avoid residual tumor tissue during surgery, the ablation boundary was enlarged by at least 2 cm according to the scope of the tumor shown in preoperative MRI. Although cooled sterilized water was applied to avoid hyperthermia of the cartilage surface during the operation, injury of the cartilage surface of the femoral head may not be totally avoided. Third, the material used to reconstruct bone defects after tumor ablation was bone cement. Although it plays a good supporting role and can reduce the probability of recurrence to some extent^23^, it also causes some damage to the cartilage during the process of hardening and heating. Finally, the tumors were located in the subfemoral head close to the articular surface in two of three cases suffering from osteonecrosis; the distance from the tumor border to the articular surface was 2.9 and 1.8 cm, respectively (and 2.3 cm in the third case). The remaining 5 cases, with tumors located in the trochanter major lower of femoral neck, did not suffering from osteonecrosis. The distance from the tumor border to the articular surface was more than 5 cm. This may also be a reason for femoral head necrosis.

### *Improvements to the Technique*

In the past 1 year, we have recently found that we can improve our technique to reduce the complications of this surgery using the following measures: (i) improve the level of preoperative cephalosporin antibiotics using the third generation of cephalosporins; (ii) minimize the time of the operation so as to reduce blood loss and incidence of infection; (iii) fill with the bone cement mixed with vancomycin, which can effectively reduce the incidence of infection; (iv) to avoid normal tissue damage caused by hyperthermia, we recommend cutting off the important ligaments before microwave ablation and then suture fixating them after the ablation procedure is completed; (v) strengthening the protection of deep tissue through complete soft tissue coverage, reducing the possibility of deep infection; (vi) for the proximal femoral lesions, the surgical technique can adopt “surgical dislocation of the hip joint,” which can protect the medial femoral circumflex artery under the premise of dislocation of the hip joint and can effectively protect the femoral head blood supply without affecting the tumor boundary^42^; and (vii) patients with tumors less than 3 cm from the articular surface are more likely to suffer from necrosis of the femoral head.

However, studies with a larger number of participants and with long‐term follow‐up research are necessary. In regard to the selection of filler after tumor ablation and curettage, we recommend filling with allogeneic bone under the osteochondral bone and the remaining tumor cavity being filled with bone cement^43^, ^44^.

### *Limitations*

The present study has some limitations. First, this research had no control group, and only a small number of patients were included in this study. Second, this is a retrospective analysis from one institution that might be limited by referral bias; thus, the evidence level is low. Despite these limitations, we considered that curettage with adjuvant microwave therapy is a safe and effective method for the management of GCTB, especially for the two types of GCTB discussed above.

### *Conclusion*

According to the results of this retrospective study, we believe that curettage with adjuvant microwave therapy is a safe and effective method to treat GCTB, especially for GCTB of the distal radius and those with pathological fracture. This therapy can effectively reduce the rate of postoperative recurrence and achieve satisfactory postoperative joint function. However, further studies with a larger number of participants and with long‐term follow‐up research need to be conducted.
